# DNA methyltransferase inhibitors combination therapy for the treatment of solid tumor: mechanism and clinical application

**DOI:** 10.1186/s13148-021-01154-x

**Published:** 2021-08-27

**Authors:** Chunhong Hu, Xiaohan Liu, Yue Zeng, Junqi Liu, Fang Wu

**Affiliations:** 1 Department of Oncology, The Second Xiangya Hospital, Central South University, Changsha, 410011 Hunan China; 2 Hunan Key Laboratory of Tumor Models and Individualized Medicine, The Second Xiangya Hospital, Central South University, Changsha, 410011 Hunan China; 3 Hunan Key Laboratory of Early Diagnosis and Precision Therapy in Lung Cancer, The Second Xiangya Hospital, Central South University, Changsha, 410011 Hunan China; 4 Hunan Cancer Mega-Data Intelligent Application and Engineering Research Centre, Changsha, 410011 Hunan China

## Abstract

DNA methylation, an epigenetic modification, regulates gene transcription and maintains genome stability. DNA methyltransferase (DNMT) inhibitors can activate silenced genes at low doses and cause cytotoxicity at high doses. The ability of DNMT inhibitors to reverse epimutations is the basis of their use in novel strategies for cancer therapy. In this review, we examined the literature on DNA methyltransferase inhibitors. We summarized the mechanisms underlying combination therapy using DNMT inhibitors and clinical trials based on combining hypomethylation agents with other chemotherapeutic drugs. We also discussed the efficacy of such compounds as antitumor agents, the need to optimize treatment schedules and the regimens for maximal biologic effectiveness. Notably, the combination of DNMT inhibitors and chemotherapy and/or immune checkpoint inhibitors may provide helpful insights into the development of efficient therapeutic approaches.

## Background

Surgery, chemotherapy and radiotherapy are the mainstays of cancer treatment. Radical operation is most often the first treatment for solid tumors. Patients for whom surgery is not an option usually receive chemotherapy and radiotherapy. Chemotherapy has limited applicability in tumor therapy because of the associated complications, including nausea, vomiting, myelosuppression and resistance. With the development of precision medicine, researchers are applying new therapies to target certain molecules within tumor cells to induce cell death. Immunotherapy has gained worldwide attention and is regarded as one of the most radical anticancer treatments to be applied to the clinic. However, immune evasion and immunosuppression complicate the immune response to tumors [1–4]. It is clear that cancer treatment has various challenges and that it is necessary to continually strive to develop new therapeutic approaches.

Epigenetics refers to inherited altered gene expression that does not involve DNA sequence alteration. Epigenetic alterations include DNA methylation, histone modification and microRNA (miRNA) alteration. Subtle epigenetic regulation controls the activity of genes to affect cancer initiation or progression [5]. Understanding the molecular mechanisms involved in the initiation and maintenance of epigenetic abnormalities in cancer has great potential for clinical translation [6].

DNA methylation is catalyzed by a group of enzymes called DNA methyltransferases (DNMTs) [7]. In mammals, the DNMT family has four members, DNMT1, DNMT3A, DNMT3B and DNMT3L. DNMT1 is required for the maintenance of methylation across the genome. DNMT3A and DNMT3B are referred to as de novo methyltransferases [8]. DNMT3L acts as a stimulator of the catalytic activity of DNMT3A and DNMT3B [9]. De novo DNA methyltransferases DNMT3A and DNMT3B in combination with DNMT3L establish a pattern of methylation that is then faithfully maintained through cell division by the maintenance methyltransferase DNMT1 [10]. DNMT alterations have been frequently observed in various types of tumors, indicating that these alterations accompany the occurrence and development of tumors [11].

DNA methylation occurs by the covalent addition of a methyl group at the 5-carbon of the cytosine ring, resulting in 5-methylcytosine formation in CpG regions, and this process is inhibited by DNMT inhibitors [12]. DNMT inhibitors activate the expression of silenced genes at low doses and are able to kill cancer cells at high doses [13, 14]. The hepatotoxicity caused by DNMT inhibitors limits their application in solid tumor treatment. However, DNMT inhibitors can be used to treat a variety of hematological tumors, including myelodysplastic syndrome (MDS), acute myeloid leukemia (AML) and chronic myeloid leukemia (CML) [15–17].

The ability of DNMT inhibitors to kill tumor cells has been acknowledged since Monparlar et al. [18] performed their seminal work, which also found that decitabine is an effective cytostatic inhibitor of tumor cells in vitro. In recent years, some studies have shown that interactions between DNMT inhibitors and chemotherapeutic drugs make combining epigenetic therapy and chemotherapy an attractive approach to circumvent the limitations of chemotherapy alone [19]. Moreover, DNMT inhibitors can reverse epidermal growth factor (EGF) receptor (EGFR) methylation, which may enhance EGFR expression and reverse EGFR tyrosine kinase inhibitor (TKI) resistance [20, 21]. Understanding epigenetics helps us to develop new mechanistic insights into pathways of immune resistance so that immunotherapy may become more widely applied as a therapeutic option in common malignancies [22, 23].

This review describes some of the most recent and promising advances in DNMT inhibitor therapy with an emphasis on the likely implications of the application of DNMT inhibitors combined with other drugs for treating solid tumors.

## DNA methylation in cancer epigenomics

In mammals, DNA methylation occurs almost exclusively in CpG regions. While 70%–80% of CpG sites are methylated, the remaining unmethylated CpG sites mostly occur in dense clusters referred to as CpG islands [24–28]. In cancer, aberrant methylation is characterized by the hypermethylation of CpG islands in tumor suppressor genes. There is a wealth of evidence that the hypermethylation of CpG islands in the promoter regions of tumor suppressor genes leads to their inactivation, and this modification is highly implicated in cancer development growth. In contrast, the upregulation of prometastatic genes induced by DNA hypomethylation promotes invasion and metastasis pathways, one of the most morbid aspects of cancer. Therefore, DNA hypermethylation and hypomethylation trigger different cellular mechanisms involved in cancer [29].

To ensure genomic integrity and stability, pericentromeric heterochromatin is highly methylated and satellite sequences and repetitive genomic sequences are silenced [30]. The loss of DNA methylation in these regions may be related to tumor development. Additionally, hypomethylated DNA may also activate latent, genome-incorporated viral sequences. For example, DNA methylation represses the expression of genital human papillomavirus (HPV) and Epstein-Barr virus proteins, which are associated with cervical cancer and nasopharyngeal carcinoma (NPC) progression, respectively [29, 30].

Methylation-associated gene silencing plays a critical role in tumor progression. Hypermethylated genes in regulatory regions are involved in a variety of important cellular pathways [30]. Taken together, these findings indicate that small noncoding RNAs and miRNAs play an important role in tumorigenesis. miRNA hypermethylation and hypomethylation frequently occur in human cancers. Understanding the cross talk between miRNAs and DNA may lead to the discovery of novel therapeutic targets [33, 34].

## DNA hypomethylating drugs and their clinical application in solid tumors

**Fig. 1:**
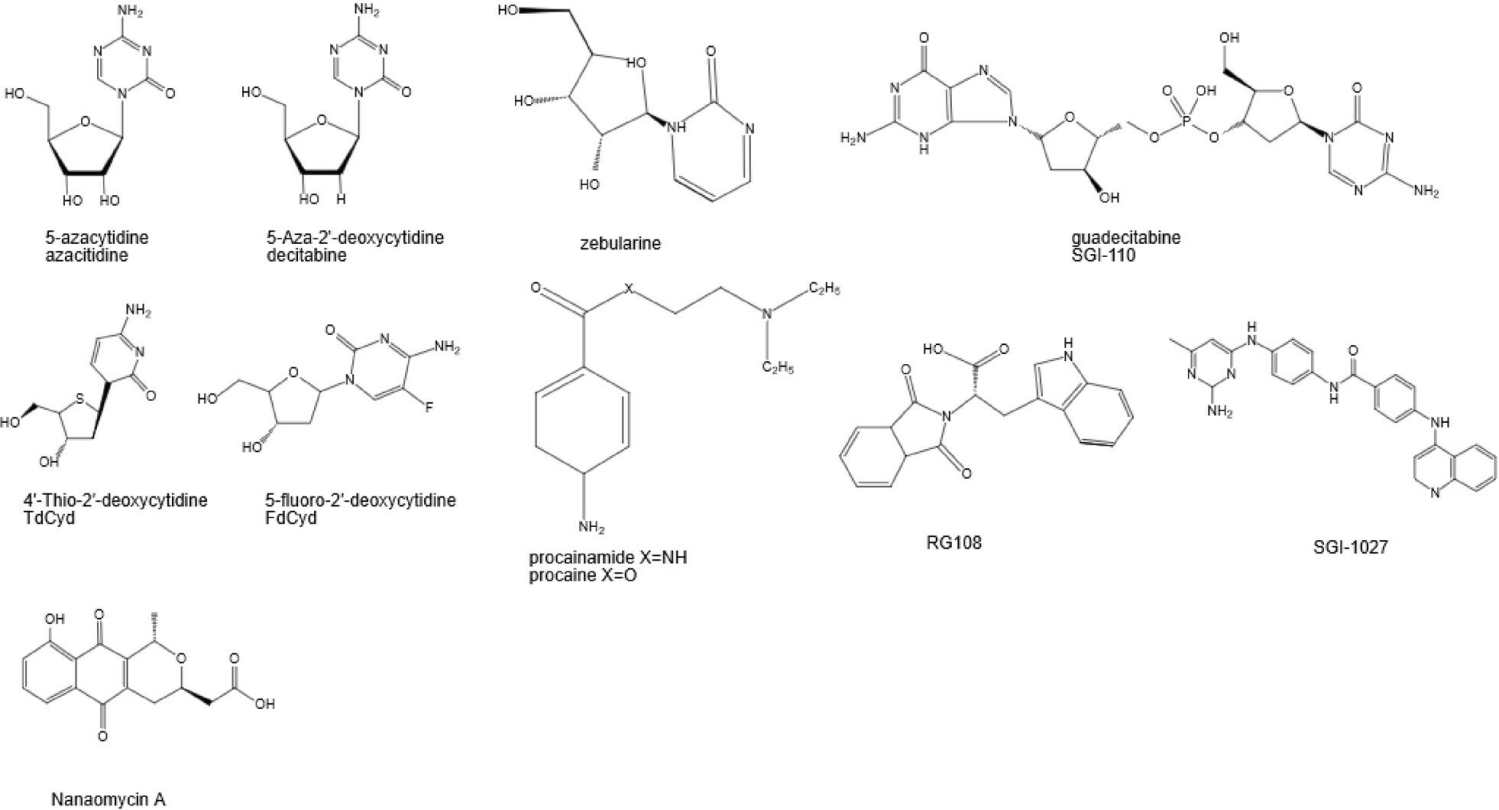
Chemical structures of nucleoside and nonnucleoside DNA inhibitors

In the early 1960s, two nucleoside DNMT inhibitors were discovered. These were 5-azacytidine (azacitidine, AZA, Vidaza) and its derivative, 5-2′-deoxycytidine (decitabine, DAC, Dacogen). Over the last several decades, the anticancer activity of these agents has been examined [35]. Recently, some new nucleoside DNMT inhibitors and nonnucleoside DNMT inhibitors, including hydralazine, procaine and MG98, have been identified and are currently being investigated as antitumor drugs (Fig. 1).

### Nucleoside analogs

Azacitidine and decitabine are the most commonly used nucleoside agents in cancer. After cellular uptake, the first limiting step is the ATP-dependent phosphorylation of nucleosides to form monophosphorylated nucleotides [36]. These monophosphorylated nucleotides are incorporated into DNA in the place of cytosine. Then, DNMTs recognize the azacytosine-guanine dinucleotide and catalyze the methylation reaction by forming a covalent bond with the cytosine ring [37]. The covalent complex at C6 cannot be resolved through b-elimination, because of the presence of a nitrogen atom at position 5. Covalently trapped DNMTs are degraded, resulting in the depletion of cellular DNMTs [36, 38]. High-dose DNMT inhibitors facilitate the formation of bulky adducts, leading to replication fork stalling and DNA replication inhibition, which causes cell death [39]. When cells are treated with low DNMT inhibitor doses, the agents are still incorporated into DNA and bind DNMTs, leading to DNMT degradation. Without DNMTs to maintain DNA methylation, CpG sites lose their methylation after cell replication, and the transcription of genes previously silenced by promoter methylation is restored [40, 41]. Decitabine can decrease DNMT1 and DNMT3A expression, reversing abnormal transcription activation, while azacitidine only targets DNMT1 [42]. Another difference between these two drugs is that azacitidine can be incorporated into both DNA and RNA, whereas decitabine can only be incorporated into DNA [18].

Azacitidine, an analog of the cytidine pyrimidine nucleoside, has received approval by the US Food and Drug Administration for the treatment of all subtypes of MDS [43]. Despite marked activity in myeloid malignancy, the use of azacitidine in patients with solid tumors is limited by toxicity, myelosuppression; and low complete and partial response rates (Table 1) [44, 45]. Recently, a two-part phase I study evaluated CC-486 (an oral formulation of azacitidine) in combination with cytotoxic agents or as monotherapy for patients with advanced solid tumors. CC-486 monotherapy resulted in partial responses (three of eight patients) and stable disease (four of eight patients) in patients with nasopharyngeal cancer. Considering the potential benefit of CC-486 as monotherapy in this study, the combination of CC-486 with immune checkpoint inhibitors could be a promising area of clinical investigation [46].

**Table 1:** Clinical trials of DNMT inhibitor monotherapies for solid tumors

Drug	Tumor types	Regiment	Phase	Patients number	Result	Year
Azacitidine	Solid tumors(breast cancer, melanoma, colon cancer)	1.0–24.0 mg/kg/day and were given over a minimal period of 8 days	I	30	SD:11/22 PD:11/22	1972
	Solid tumor (breast cancer and other carcinoma)	1.6 mg/kg/day on days 1–10 and followed by a maintenance regimen	II	148	–	1977
CC-486	Relapsed or refractory solid tumors	300 mg/day (oral) on days 1–14 and day 21	I	20	PR:3/8 SD:4/8	2018
Fazarabine	Refractory metastatic colon cancer	2 mg/m^2^/h, continuous infusion 72 h every 3–4 weeks	II	18	–	1993
	Refractory solid tumors	30 mg/m^2^, daily bolus 5 times	I	–	–	1993
Decitabine	Metastatic solid tumors	20–40 mg/m^2^/day continuous infusion on days 1–3 (28-day cycle)	I	19	PD:14/14	2003
	Solid tumors (ovarian, renal, breast, colon)	2 mg/m^2^/day 7-day continuous infusion (28-day cycle)	I	10	SD:2/9 PD:7/9	2005

Decitabine is a unique cytosine analog and has recently emerged as a therapy for MDS and CML. Although the promise of these hypomethylating drugs has not been realized for solid tumor cancer therapy, researchers contend that decitabine can achieve optimal biological effects at low doses [47]. In the 2000s, decitabine monotherapy produced unsatisfactory results for patients with solid tumors [48, 49]. Ten patients with refractory solid tumors were included in a phase I study, where decitabine was administered via continuous infusion at 2 mg/m^2^/day for 168 h. After the treatment cycles, no objective responses were observed, and seven of ten patients exhibited disease progression after one or two cycles. Samlowski et al. [49] examined the expression of select genes after the start of treatment, and their results showed MAGE-1 promoter hypomethylation.

Zebularine is a cytidine analog that lacks the amino group at position 4 of the pyrimidine ring. Zebularine has high stability and low toxicity, and it is stable at acidic and neutral pHs, enabling oral administration [37, 50]. When zebularine traps DNMT on DNA, zebularine becomes an obstacle for the second round of replication. This results in a collapsed replication fork and the formation of replication-dependent double stand breaks (DSBs) [51]. Moreover, zebularine can suppress the interaction of DNMT1 with G9a histone methyltransferases, which may regulate the survival and apoptosis of human cancer cells [52]. Transient zebularine exposure produces differential cell density-dependent responses and correlates with the overexpression of genes related to cancer stem cells and the key epithelial–mesenchymal transition process [53]. Although zebularine is more stable and less toxic than azacitidine and decitabine, clinical trials are required to demonstrate its therapeutic effect in solid tumors.

Guadecitabine (SGI-110) is a second-generation decitabine and deoxyguanosine compound with prolonged half-life and activity in AML and high-risk AML. Guadecitabine addresses the shortcomings of first-generation DNMT inhibitors that are susceptible to deamination by cytidine deaminase (CDA). CDA is found in multiple organs in the body, causing first-generation DNMT inhibitors to have short plasma half-lives. Guadecitabine has improved stability that confers enhanced DNA incorporation into dividing cells and is more resistant to CDA [54]. Based on these factors, it is believed that guadecitabine may be a more appropriate DNMT inhibitor than azacitidine and decitabine [55, 56]. Guadecitabine has been demonstrated to have clinical activity in MDS and AML [57, 58]. However, a substantial difference in cost in combination with a marginal difference in survival benefit might limit its use in the clinical setting [59].

Another cytosine analog, 4′-thio-2′-deoxycytidine (TdCyd), has been used in clinical trials for patients with advanced solid tumors [60]. This compound incorporates into the DNA sequence recognized by the bacterial C5 DNA methyltransferase M. 5-Fluoro-2′-deoxycytidine (FdCyd) has also been assessed in clinical trials for the treatment of advanced solid tumors, AML and multiple sclerosis (MS) [61, 62]. In both in vitro and in vivo models, TdCyd and FdCyd potently deplete DNMT1 in cancer and concomitantly inhibit tumor growth [63].

### Nonnucleoside analogs

To overcome the disadvantages of nucleoside analogs, including poor bioavailability, chemical instability under physiological conditions and a lack of selectivity, nonnucleoside analogs have been developed over the last decades [64]. The structures of nonnucleoside analogs are very heterogeneous, but their mechanisms of action are independent of DNA incorporation. Some drugs (including procainamide, an amide, and its ester analog procaine) have been repurposed after they were shown to have demethylating effects. These agents show affinity for CpG-rich regions of DNA, blocking the activity of DNMTs and reactivating some tumor suppressor genes [65].

SGI-1027 was synthesized as a quinoline-based compound and was described for against DNMT1, DNMT3A and DNMT3B [66, 67]. After that, Valente et al. [68] and Rilova et al. [67] described two analogues of SGI-1027, which are MC3343 and MC3353. SGI-1027 and its analogue share DNA-competitive and AdoMet non-competitive behavior on DNMT1 [64]. SGI-1027 may inhibit DNMT activity, induce the degradation of DNMT1 and reactivate tumor suppressor genes [69]. SGI-1027 can also impair cervical cancer cell and hepatocellular carcinoma cell propagation by dramatically increasing apoptotic cell death and cell cycle arrest [69, 70]. As a novel DNMT inhibitor, MC3343 is more potent and selective than SGI-1027 toward other S-adenosylhomocysteine-dependent (SAM-dependent) methyltransferases [71, 72]. Zwergel et al. reported that MC3353 displays a stronger in cell demethylating ability than both azacitidine and decitabine. Besides, this compound proved antiproliferative activity in several cancer cell line types [73].

In addition to SGI-1027, some oligonucleotides are accommodated in the catalytic pocket of DNMTs, where they effectively function as competitive inhibitors. MG98 has shown interesting preclinical evidence that it can inhibit DNMT1 [74], allowing for the re-expression of tumor suppressor genes and tumor growth inhibition [75, 76]. In an open-label phase I study, patients with advanced solid malignancies were treated with escalating MG98 doses administered as a continuous infusion over 7 days repeated every 14 days. After two cycles, suppression of DNMT1 expression was observed in 26 of the 32 patients studied. One patient achieved a partial response, and another achieved prolonged disease stabilization [76].

N-Phthaloyl-L-tryptophan (RG108), a DNMT1 inhibitor [77], targets DNMT1 SAM cofactor binding. RG108 significantly inhibits the proliferation of endometrial cancer cells, blocks the cell cycle in the G2/M phase and induces apoptosis by increasing hMLH1 expression and inhibiting DNMT3B expression [78]. Selective nonnucleoside DNMT1 inhibitors in the DC_05 series of compounds can also play an anticancer role by inducing DNA hypomethylation to restore tumor suppressor gene expression [40]. Interestingly, the histone H3 lysine 9 methyltransferases (G9a/GLP) inhibitor BIX-01294 showed novel ability to inhibit the DNA methyltransferase DNMT3A at low micromolar levels without inhibition of DNMT1 and G9a [80]. Nanaomycin A is the first selective DNMT3B inhibitor that can induce genomic demethylation. Nanaomycin A interacts with DNMT3B amino acid residues that are involved in methylation, preventing DNMT3B from participating in normal DNA methylation [81]. Nanaomycin A treatment reduces global methylation levels in cancer cell lines and reactivates transcription of the RASSF1A tumor suppressor gene [82].

## Experimental studies on the effects of combination therapies using DNMT inhibitors

### Enhanced radiation sensitivity

Kumar et al. [83] examined γ-radiation-resistant and γ-radiation-sensitive cell lines to determine the relationship between radiation sensitivity and DNA methylation. They demonstrated that treating cells with decitabine and trichostatin A (TSA) before irradiation enhanced DNA strand breakage, G2/M phase arrest, apoptosis and cell death. Moreover, γ-radiation increased the transcriptional activity of the p16^INK4a^ and ATM gene promoters by altering DNA methylation levels. ^111^Indium-labeled human epidermal growth factor (^111^In-DTPA-hEGF) is an auger electron-emitting agent that targets EGFR-overexpressing cells. Together with (111) In-DTPA-hEGF, decitabine can sensitize breast cancer to ionizing radiation and induce DNA destruction [84]. Kim et al. [85] investigated the underlying cellular mechanisms of combination treatment using ionizing irradiation and decitabine in human colon cancer cells. After this treatment, colon cancer cell growth was significantly lower than that with decitabine or radiotherapy alone, and increases in the number of G1-phase cells and the apoptosis rate were observed for colon cancer cells. Recently, Ou et al. [86] found that RG108 increased the radiosensitivity of esophageal cancer cells. Esophageal cancer cell apoptosis and G2/M-phase arrest were induced by X-ray irradiation and were significantly enhanced by RG108.

### Increased sensitivity to anticancer drugs

**Fig. 2:**
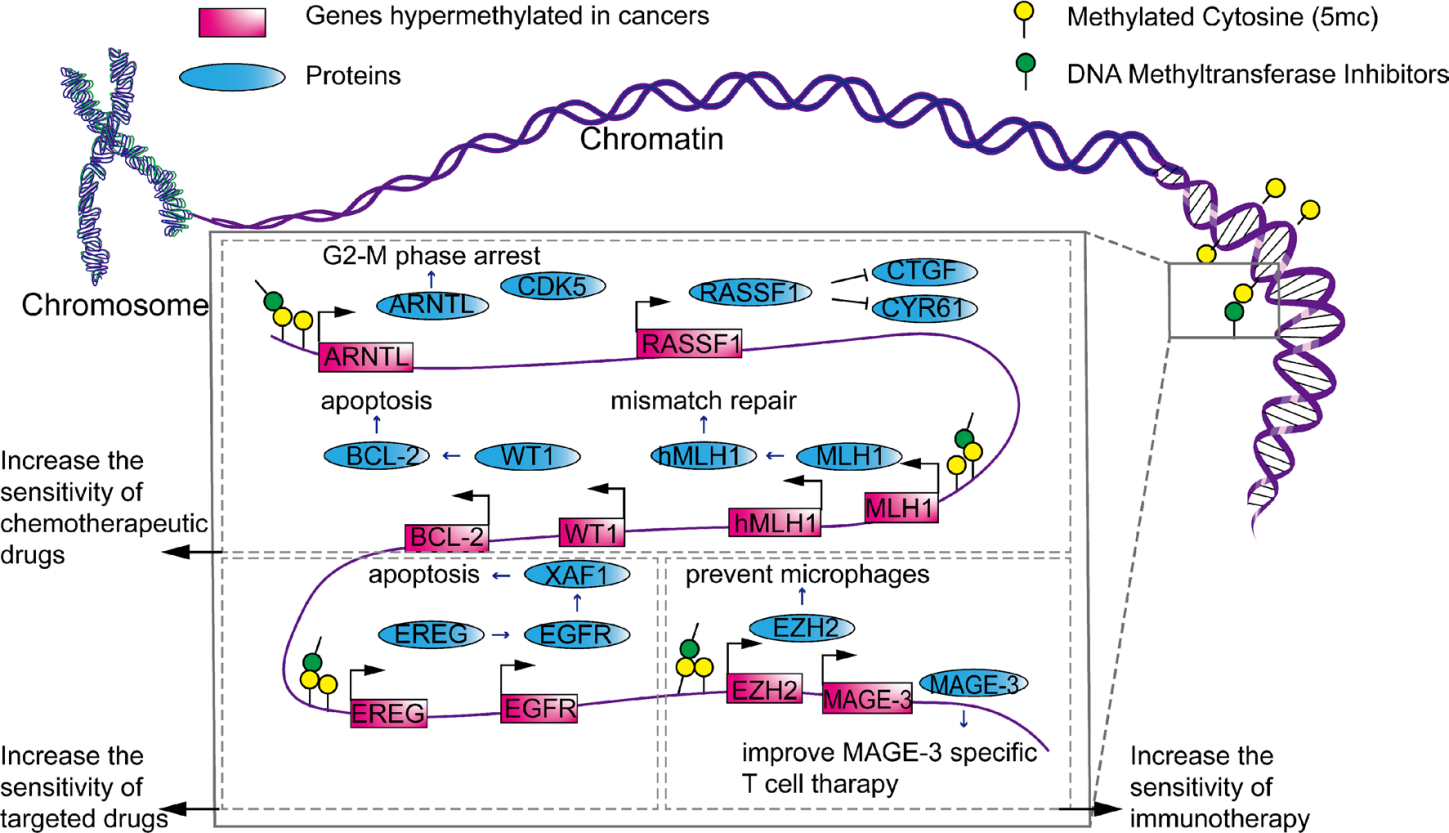
Molecular regulatory mechanisms of DNMT inhibitors in increasing the sensitivity to drugs. DNMT inhibitor treatment can increase the sensitivity of chemotherapeutic drugs via the methylation status of ARNTL, RASS1, MLH1, hMLH1, WT1 and BCL-2. DNMT inhibitors are able to sensitize tumor-targeting drugs through the induction of various proteins, such as EREG, EGFR and XAF1. They can also enhance immunotherapy by targeting EZH2 and MAGE-3

In the 1990s, Fost. et al. [87] explored the combined use of decitabine and cisplatin in vitro. They demonstrated the synergistic cytotoxicity of this drug combination against a panel of six human cell lines. Epigenetic priming with decitabine can improve the sensitivity of gastric cancer cells to SN38 (doxorubicin) and cisplatin [88]. Low-nanomolar doses of decitabine and azacitidine induce sustained antitumor responses [89]. In myeloma cell lines, researchers observed a significant phenomenon of cell proliferation inhibition after combination therapy of decitabine with adriamycin [90]. Several studies have investigated the molecular mechanisms through which DNMT inhibitors affect the efficacy of other drugs (Fig. 2).

A comparative study showed that platinum-resistant cell lines exhibited more epigenetic alterations than platinum-sensitive cell lines, and the hypermethylation of promoter regions was significantly increased. The authors identified 14 genes that were hypermethylated in cisplatin-resistant cell lines but not in cisplatin-sensitive parental cell lines. Six of 14 genes (SAT, C8orf4, LAMB3, TUBB, G0S2 and MCAM) were cisplatin inducible in sensitive cell lines but not in resistant cell lines [91]. DNMT inhibitors demethylated the promoter CpG regions of *ARNTL*. The ARNTL protein suppressed NPC cell proliferation and enhanced cell sensitivity to cisplatin by targeting CDK5. *ARNTL* overexpression suppressed NPC cell proliferation in vitro and in vivo, and the opposite effect was observed following *ARNTL* silencing. Gene set enrichment analysis (GSEA) revealed that ARNTL is associated with the cell cycle and that ectopic expression and overexpression of ARNTL could induce G2-M phase arrest [92]. Moreover, in an in vivo melanoma model, DNMT inhibitors augmented the hypermethylation status of the *RASSF1* gene promoter, targeted the *CTGF* and *CYR61* genes through the hippocampal pathway and increased the sensitivity of bladder cancer cells to cisplatin and adriamycin [15]. Moreover, MLH1 expression was closely related to the methylation status of the hMLH1 promoter [93]. Ovarian cancer cell lines showed increased hMLH1 promoter methylation after developing drug resistance, and a correlation was observed between *hMLH1* methylation and the general survival rate (*p* < 0.01) [94]. Several studies have shown that decitabine can reverse cisplatin resistance by inhibiting *hMLH1* in human non-small cell lung cancer (NSCLC) and esophageal carcinoma [95, 96]. DNMT inhibitors augment the sensitivity of tumor cells to irinotecan drugs (CPT11/SN38) by targeting the *BCL-2* oncogene and increasing BCL-2 protein expression [97, 98]. VHL-TGFBI hypomethylation was found to be related to the sensitivity to paclitaxel (PTX) [99].

Methylation of the *EGFR* promoter inhibits *EGFR* expression in a variety of tumor cells. Three NSCLC cell lines (H1650, H1299 and PC-9) with different EGFR mutation statuses and levels of EGFR-TKI sensitivity were used in this study. The results showed that the *EGFR* promoter region was unmethylated in PC-9 cells and that these cells were sensitive to gefitinib (an EGFR-TKI drug). In contrast, the *EGFR* promoter region was methylated in H1650 and H1299 cells, and the cells were resistant to gefitinib [100]. Treatment with decitabine resulted in the re-expression of *EGFR* in CAMA1 and MB453 cell lines, which are relatively resistant to gefitinib. However, after cotreatment with decitabine and gefitinib, a significant effect was observed on apoptosis induction. DNMT inhibitors can reverse the hypermethylation status of EGFR promoters in different cancers, which may enhance *EGFR* expression and reverse EGFR-TKI resistance [20, 21, 101]. Qu et al. [102] confirmed that upregulated EGFR expression through promoter demethylation was associated with the adenoma–carcinoma transition, and this was accompanied by an increase in EGFR phosphorylation, as assessed by reverse-phase protein analysis. Jiyoeu et al. [103] found that the hypomethylation of epidermal regulatory protein (EREG) binding with EGFR-induced gastric cancer cells grew. *DNMT3b* knockdown significantly increased EREG expression and did not significantly affect EREG promoter methylation. In another study, combined treatment with decitabine and gefitinib increased XIAP-associated factor 1 (XAF1) expression, which plays an important role in apoptosis [104].

Significantly tumor growth inhibition and prolonged survival were observed in the CT26 mouse model after treatment with a combination of PD-1 blockade and decitabine versus treatment with decitabine or PD-1 blockade alone. Decitabine may provide clinical benefits to patients with colorectal cancer and low microsatellite instability or microsatellite stability [105]. In NSCLC, combining the DNA hypomethylating agent azacytidine with anti-PD-1 therapy significantly reduced tumor size compared with that with anti-PD-1 therapy alone. This combination might therefore be a promising approach to overcoming anti-PD-1 resistance [106].

### Identification of biomarkers

High levels of methylated *CFTR* are observed in breast cancer, and *CFTR* overexpression can inhibit breast cancer cell growth. Increased cell invasion was observed following *CFTR* knockdown. These results suggest that *CFTR* might be a diagnostic marker of breast cancer [107]. DACT2 is frequently inactivated by CpG methylation in NPC. DNMT inhibitors inhibit NPC cell proliferation and metastasis through the suppression of β-catenin/Cdc25c signaling. A study suggested that DACT2 promoter methylation was a potential epigenetic biomarker for the detection of NPC and for chemotherapy guidance [96]. Stewart et al. [98] showed that KRAS genomic status predicted decitabine sensitivity in ovarian cancer cells. Pretreatment with decitabine decreased the cytotoxic activity of MEK inhibitors in KRAS-mutant ovarian cancer cells, with reciprocal downregulation of DNMT1 and MEK/ERK phosphorylation. This study implicated KRAS status as a biomarker of drug response in ovarian cancer. BRAF^V600e^ plays an important role in melanoma tumorigenesis. Hou et al. [109] investigated the role of BRAF^V600E^ signaling in altering gene methylation in the genome of melanoma cells and identified genes coupled to BRAF^V600E^ signaling through examination of methylation aberrations. The results indicate that a wide range of genes with broad functions are linked to BRAF^V600E^ signaling through hypermethylation or hypomethylation. Low-dose decitabine therapy promotes antitumor T cell responses by promoting T cell proliferation, and an increased proportion of IFNγ + T cells may act as a prognostic biomarker of the decitabine-based antitumor therapy response [110].

### Cancer cell reprogramming

Low-dose decitabine treatment remarkably enhanced the effects of cisplatin and gemcitabine on basal-like bladder cancer in vivo and in vitro. These effects were accompanied by decreases in genome-wide DNA methylation, gene re-expression and changes in key cellular regulatory pathways, including STAT3 signaling [111]. DNA methylation status sequencing at different time points during colitis-associated cancer (CAC) revealed that 811 genes were hypermethylated at different time points during CAC initiation and progression. These hypermethylated genes, including *BAD* and inositol polyphosphate phosphatase-like 1 (*INPPL1*) hub genes, are involved in the MARK and EGF/EGFR pathways [112]. Tumor growth and drug response were assessed in PANC-1 cells (pancreatic ductal adenocarcinoma, PDAC) after exposure to a noncytotoxic dose of azacitidine. The authors observed that unique peptides (SST and SSTR2) were expressed in the pancreas and confirmed that azacitidine epigenetically reprogrammed PANC-1 cells to induce anticancer effects [113].

DNMT inhibitors promoted *MIG-6* re-expression by inhibiting *MIG-6* promoter methylation. The negative feedback of *MIG-6* expression increased the number of EGFR receptors [114]. Chou-Talalay analysis showed that, in bladder cancer cells, the combination of decitabine with an entinostat (ENT) histone deacetylase inhibitor could not reverse chemoresistance. However, the combination treatment between decitabine and ENT led to forkhead box class O1 (*FoxO1*) upregulation, and *FoxO1* expression resulted in increased relapse-free survival in patients with bladder cancer. Moreover, this combination further activated proapoptotic Bim and p21, cell cycle regulators [115]. These results show that low *FoxO1* expression in tumor specimens may be associated with resistance to cisplatin first-line therapy in patients with bladder cancer.

### Eliciting an immune response against cancer

**Fig. 3:**
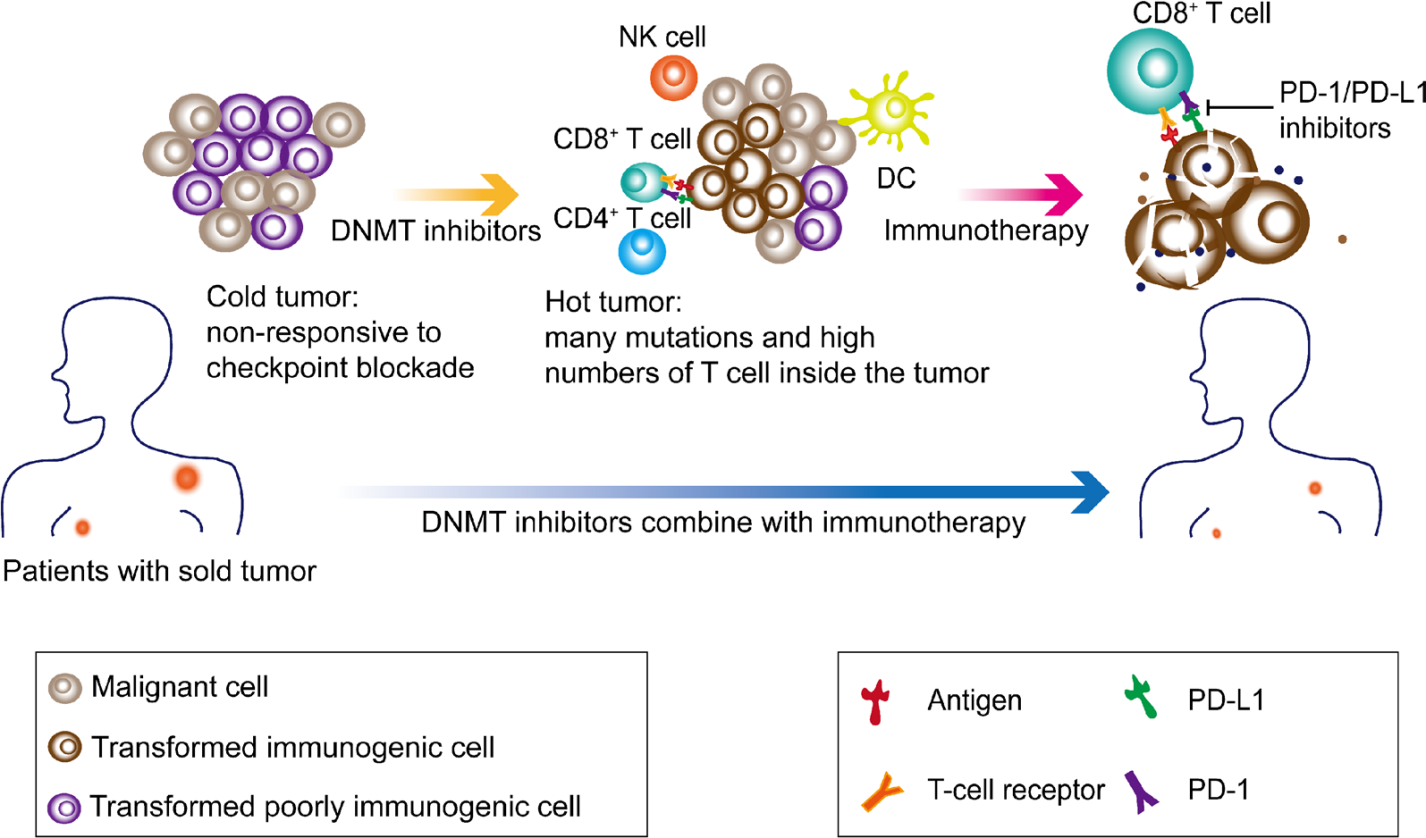
DNMT inhibitors in immune-oncology

The immune system maintains the function of the body when attacked by external substances through its two roles as a "monitor" and "protector" [116, 117]. Deregulated immune systems cannot effectively kill tumor cells, leading to immune evasion [118]. There is evidence that tumor immune evasion is mediated by nonmutational epigenetic events involving chromatin and that epigenetics and mutations collaborate to determine the state of tumor progression. Therefore, epigenetic therapy has become a “double-edged sword” with potential value in immune therapy (Fig. 3) [119, 120].

Although human endogenous retroviral sequences (ERVs) make up approximately 8.5% of our DNA, they have not been extensively studied because their repetitive nature complicates mapping [121]. Several studies have highlighted the importance of DNA methylation in the suppression of ERVs [122]. It is possible that DNMT inhibitors can reactive ERVs. After reactivation, repeat elements produced by ERVs may form nucleic acid molecules of various configurations that are then sensed by the innate immune machinery to trigger an immune response [120].

Decitabine treatment may result in the production of the antigen encoded by *MAGE-1* (a cancer testis antigen (CTA) member). *MAGE-1* is associated with major histocompatibility complex class I molecules at the cell surface for T-cell recognition [123]. Thus, CTAs are a potential source of new tumor cell surface antigens and are widely used in CAR T cell production [124, 125]. The efficacy of coupling an immune checkpoint blockade approach with a DNMT inhibitor may be increased by taking advantage of a bystander effect by attracting T cells to the tumor and simultaneously enforcing the uniform expression and display of CTAs [120].

Decitabine treatment enhances human IFNγ + T cell activation and proliferation and promotes Th1 polarization and the activity of cytotoxic T cells in vivo and in vitro. DNA hypomethylation directly enhances *PD-L1* expression in tumor cells and increases the expression of immune-related genes and T cell infiltration [110]. Overexpression of *DNMT1* and *EZH2* can result in the consumption of B cells and prevent macrophage production. This may explain why decitabine can increase the antitumor T cell response [126]. In another study, Peng et al. announced that DNMT inhibitors may improve the clinical efficacy of MAGE-A3-specific T cell therapy by increasing target gene expression [127].

## Clinical findings on DNMT inhibitor combination therapy in solid tumors

### Combinations with platinum-based chemotherapy

The majority of combination DNMT inhibitor therapies assessed to date have involved the combination of decitabine and platinum drugs. We collected decitabine-based clinical trials from the National Center for Biotechnology Information (NCBI) database in April 2021 (Table 2).

**Table 2:** Clinical trials of decitabine-based therapies for solid tumors

Drugs used in combination with decitabine	Regimen	Tumor types	Phase	Patient number	Results	Major toxicity	Response pulse stable disease rate (%)	Year
Cisplatin	DAC in four (I–IV) dose escalation levels (45, 67, 90 to 120 mg/m/day 1–3) infusion followed by cisplatin 33 g/m^2^/day 1 infusion (21-day circle)	Non small cell lung cancer (NSCLC)	I/II	14	PD:14/14	Neutropenia and thrombocytopenia	0	2000
Cisplatin	DAC 50 mg/m^2^/day followed by cisplatin 30 g/m/day 3-h infusion (21-day cycle)	Advanced cervical cancer(most of them are squamous cell carcinoma of the cervix)	II	25	PR:8/21 SD:5/21 PD:8/21	Hematologic toxicity	62	2002
Carboplat	DAC 90 mg/m^2^ on day1 6-h infusion followed by carboplat in on day8 (28-days circle)	Solid tumors (colon, breast, ovary, melanoma, sarcoma, gall bladder and pleural mesothelioma)	I	35	PR:1/10 SD:3/10 PD:6/10	Myelosuppression	40	2007
Carboplat	DAC (10 or 20 mg/m^2^/day1-8 and carboplatin on day 8 (28-day cycle)	Platinum-resistant ovarian cancer	I	10	CR:1/10 SD:6/10 PD:3/10	Nausea, allergic reactions and neutropenia	70	2010
Carboplat	DAC 10 mg/m^2^ for day1-5 and carboplatin on day 8 (28-day cycle)	Platinum-resistant ovarian cancer	II	17	CR:1/17 PR:5/17 SD:6/17 PD:5/17	Nausea	70	2012
Carboplat	DAC 90 mg/m^2^ day1 infusion followed by carboplat infusion on day 8 (28-days circle)	Relapsed ovarian cancer	II	15	PR + SD:1/12 PD:11/12	Neutropenia	8	2014
Carboplat	DAC 7 mg/m^2^ day 1–5 followed by reduced TC treatment (28-day circle)	Relapsed or refractory ovarian cancer	I/II	21	PR:3/17 SD:9/17 PD:5/17	Nausea and neutropenia	71	2015
Carboplat	DAC 7 mg/m^2^ day 1–5 followed by reduced TC treatment on day 6 (28-days circle)	Recurrent ovarian cancer	I/II	40	CR:1/40 PR:8/40 SD:19/40 PD:12/40	Nausea	74	2017
Doxorubicin and cyclophosphamide	DAC (5–10 mg/m^2^/day 1–7 1-h infusion followed by doxorubicin (45 mg/m) and cyclophosphamide (1 g/m) on day 7	Children refractory solid tumors (neuroblastomar,habdomyosarcoma, osteosarcoma)	I	21	SD:7/21 PD:14/21	Neutropenia and thrombocytopenia	33	2010
Vorinostat	DAC 10 mg/m^2^/day1-5 and vorinostat 200 mg twice a day on days6-12 (28-days circle)	Advanced solid tumors and non-Hodgkin’s lymphomas	I/II	43	SD:11/38 PD:27/38	Neutropenia and thrombocytopenia	29	2011
anti-EGFR	DAC 45 mg/m^2^/day 1 and 15 and followed by anti-EGFR 6 mg/kg /day 8 and 22 (28-day cycle)	wt KRAS metastatic colorectal cancer	I/II	20	PR:2/20 SD:10/20 PD:8/20	Rash and hypomagnesemia	60	2013
Vemurafenib	3 + 3 dose escalation combining subcutaneous decitabine at different doses and schedules (4 cohorts) with the standard oral dose of vemurafenib 960 mg twice daily	Metastatic melanoma	I/II	14	CR:3/14 PR:3/14 SD:5/14 OD:3/14	Fatigue and increased creatinine	79	2017

In 2000, Schwartsmann et al. designed a clinical trial using a fixed dose of 33 mg/m^2^ cisplatin and four escalating doses of decitabine (45, 67, 90 and 120 mg/m^2^). However, only a short-lasting partial response was observed in a single patient with cervical cancer, and two minor responses were documented in patients with NSCLC and cervical cancer [128]. Pohlmann et al. also reported the administration of a decitabine-based combination in 2003. Patients with advanced cervical cancer received decitabine (50 mg/m^2^/day) during a 3-h continuous infusion on day 1, which was followed by the administration of cisplatin (33 g/m^2^/day) on day 4 of a 21-day cycle. Evaluation after 2 cycles revealed a satisfactory response rate, with eight patients (38.1%) achieving a partial response and five patients (23.8%) achieving stable disease [129].

Patients with ovarian cancer are often administered a platinum compound and a taxane. Several phase I or phase II clinical trials used a low dose of decitabine combined with carboplatin to treat platinum-resistant ovarian cancer or relapsed ovarian cancer (Table 2) [130–135]. Among those regimens, a clinical trial administered decitabine (7 mg/m^2^/day) on days 1–5 followed by reduced taxane and carboplatin. This approach achieved an effective clinical response, with nine patients (22.5%) achieving complete or partial response and nineteen patients (47.5%) achieving stable disease. Notably, *MLH1, RASSF1A, HOXA10 and HOXA11* demethylation in tumors was positively correlated with progression-free survival (*p* < 0.05). Low-dose decitabine altered gene DNA methylation and cancer pathways, restored carboplatin sensitivity in patients with heavily pretreated ovarian cancer and resulted in a high objective response rate and prolonged progression-free survival [132].

### Combinations with other chemotherapeutic drugs

A phase I clinical trial recruited pediatric patients with solid tumors. These patients were treated with decitabine (5 mg/m^2^/day) during a 1-h continuous infusion on days 1–7 and then with doxorubicin (45 mg/m^2^/day) and cyclophosphamide (1 g/m2/day) on day 7. In total, 60% (12/20) and 87.5% (14/16) of patients displayed significant *MAGE-1* and *HbF* demethylation, respectively, in peripheral blood mononuclear cells [136]. In another phase I study, Stathis et al. studied different doses of decitabine and vorinostat (six sequential and three concurrent doses). The maximum tolerated dose on the sequential schedule was 10 mg/m^2^/day decitabine on days 1–5 and 200 mg vorinostat three times a day on days 6–12. The results showed that 11 of the 38 patients with solid tumors and non-Hodgkin's lymphoma had a stable response after four treatment cycles [137].

### Combinations with molecular targeted therapy

Garrido-Laguna et al. conducted a phase I study to evaluate decitabine in combination with panitumumab (an antibody against EGFR) in wild-type KRAS metastatic colorectal cancer (mCRC) patients. Two of 20 patients (10%) had a partial response, but both had previously received cetuximab and another treatment. Ten patients had stable disease (three of them had stable disease longer than 16 weeks). Decreased *MAGE* promoter methylation was not observed in peripheral blood mononuclear cells [138].

The *BRAF* gene regulates the methylation of a wide number of genes and affects multiple cellular functions [109]. A phase Ib study used 3 + 3 dose escalation combining different doses and schedules of subcutaneous decitabine administration with the standard oral dose of vemurafenib (960 mg) twice daily. Fourteen ^V600^^E^BRAF-positive patients with metastatic melanoma were placed into four groups, and each group received a different regimen. Three patients achieved a complete response, three had a partial response, and five had stable disease. Preclinical assessment demonstrated that this combination treatment delayed the development of acquired resistance and improved the duration of treatment sensitivity [139].

### Combinations with immunotherapy

In NSCLC, immunotherapy produced an astounding result. An objective response (a complete or partial response) was observed in 5 of 49 patients with NSCLC. These patients passed the 24-week point without progression with subsequent immune checkpoint therapy, and three of the five developed high-grade partial responses (according to the Response Evaluation Criteria in Solid Tumors (RECIST)) that remained durable over 2.5 years [140, 141]. Eighty-six anti-PD-1 treatment-naïve patients were randomly assigned (1:2) to camrelizumab (200 mg) monotherapy or decitabine (10 mg/d, days 1–5) plus camrelizumab (200 mg, day 8) combination therapy administered every 3 weeks. At the time of analysis, the response duration rates of camrelizumab monotherapy and decitabine plus camrelizumab combined therapy at 6 months were 76% and 100%, respectively. The complete response rate was 32% (6 of 19 patients) with camrelizumab monotherapy versus 71% (30 of 42 patients) for those administered decitabine plus camrelizumab (*p* = 0.003). Researchers concluded that decitabine plus camrelizumab may reverse the resistance to PD-1 inhibitors in patients with relapsed/refractory classical Hodgkin lymphoma (cHL) [142].

Two different clinical trials combined decitabine and cytokine-induced killer (CIK) cells. The first study divided 52 recurrent ovarian cancer patients with platinum resistance into two groups. Patients in the paclitaxel and carboplatin (DTC) group were treated with decitabine and a reduced dose of paclitaxel and carboplatin. Patients in the DTC + CIK cell group were treated with the same regimens and received CIK cell therapy. Notably, DTC + CIK cell treatment in platinum-resistant/refractory patients led to an overall response rate of 87.50%, a progression-free survival tome of 8 months and an overall survival time of 19 months. DTC treatment in platinum-resistant/refractory patients led to an overall response rate of 22.5%, a progression-free survival time of 4 months and an overall survival time of 12 months. These data indicate that decitabine might show a remarkable clinical response when combined with adoptive immunotherapy in patients with platinum-resistant/refractory ovarian cancer [143]. Another clinical trial enrolled 45 patients with drug-resistant relapsed/refractory esophageal, gastric or colorectal cancers. Patients received decitabine on days 1–5 and were then divided into two groups. Some patients were treated with previous chemotherapy (the DC cohort), while others received CIK cell therapy after previous chemotherapy (DC + CIK cell cohort). In the DC cohort, patients had an overall response rate of 20%, a disease control rate of 70%, a progression-free survival time of 4 months and an overall survival time of 12 months. However, in the DC + CIK cell cohort, the patients had an overall response rate of 28%, a disease control rate of 92%, a progression-free survival time of 6 months and an overall survival time of 11 months [144]. The toxicity and overall response rate observed did not significantly differ between cancer types and treatment cohorts.

## Conclusion

The mechanism by which DNMT inhibitors function in combination with antitumor drugs has not yet been fully elucidated. However, the studies explored in this review show that, in most cases, combination treatment with DNMT inhibitors and antitumor drugs has higher efficacy than treatment using antitumor drugs alone. However, there are many hurdles to overcome before the routine clinical application of this therapeutic approach. The sample size for clinical trials is small, with most studies involving fewer than 50 patients. Moreover, there are very few studies that use randomized, blind, controlled designs.

Although combination treatments using DNMT inhibitors and antitumor drugs may provide helpful insights into the development of efficient therapeutic approaches for cancer treatment, further investigation is needed. Such studies should include randomized controlled trials with large sample sizes.

## Data Availability

Data sharing is not applicable to this article as no datasets were generated or analyzed during the current study.

## Abbreviations

DNMT: DNA methylation transferase
miRNA: MicroRNA
EGFR: Epidermal growth factor receptor
NPC: Nasopharyngeal carcinoma
MDS: Myelodysplastic syndrome
CML: Chronic myeloid leukemia
AML: Acute myeloid leukemia
TKI: Tyrosine kinase inhibitor
AdoMet: S-adoenosyl-L-methionine
SAM: S-adenosylhomosteine
TSA: Trichostatin A
NSCLC: Non-small cell lung cancer
RR: Response rate
PFS: Progression-free survival
CTAs: Cancer testis antigen
INPPL1: Inositol polyphosphate phosphatase-like 1
CR: Complete response
PR: Partial response
SD: Stable disease
PD: Progressive disease
